# Somatic Embryogenesis and Plant Regeneration in *Viola canescens* Wall. Ex. Roxb.: An Endangered Himalayan Herb

**DOI:** 10.3390/plants10040761

**Published:** 2021-04-13

**Authors:** Arun Kumar Khajuria, Christophe Hano, Narendra Singh Bisht

**Affiliations:** 1 Plant Tissue Culture Laboratory, Department of Botany, Campus Pauri, HNB Garhwal University, Pauri Garhwal 246001, Uttarakhand, India; nsbotany@gmail.com; 2 Department of Botany, Cluster University of Jammu, Jammu 184001, Jammu & Kashmir, India; 3 Laboratoire de Biologie des Ligneux et des Grandes Cultures (LBLGC), INRAE USC1328, Campus Eure et Loir, Université d’Orléans, F-28000 Chartres, France; hano@univ-orleans.fr; 4 Bioactifs et Cosmétiques, Centre National de la Recherche Scientifique (CNRS) GDR3711, Université d’Orléans, CEDEX 2, F-45067 Orléans, France

**Keywords:** callus, casein hydrolysate, silver nitrate, abscisic acid, cytokinin, survival

## Abstract

*Viola canescens* Wall. ex. Roxb. is an important but threatened medicinal herb found at 1500–2400 m above mean sea level in the Himalayas. Overexploitation and habitat preference have put the plant under serious threat. Thus, the present study was undertaken to develop an efficient protocol for in vitro propagation via somatic embryogenesis. The results revealed that plant can be regenerated successfully through somatic embryogenesis using leaf derived calli. Regular subculturing of calli on Murashige and Skoog (MS) medium with 2,4-dichlorophenoxyacetic acid (2,4-D)/indole-3-butyric acid (IBA)/kinetin (Kn) and varying combinations of 2,4-D+Kn induced somatic embryogenesis. The maximum average number of somatic embryos (SE) (19.15 ± 2.66) was induced on the medium with 0.15 + 0.05 mg L^−1^ of 2,4-D and Kn, respectively, and this medium was used as a control. To enhance somatic embryo induction, the control MS medium was supplemented with l-glutamine (200–400 mg L^−1^) and casein hydrolysate (1–4%). The maximum average number of SE (27.66 ± 2.67) and average mature SE (13.16 ± 3.48) were recorded on the medium having 2 % l-glutamine and 50 mg L^−1^ casein hydrolysate. The induced SE were asynchronous, so, to foster their maturation, the culture medium (free from growth regulators) was supplemented with abscisic acid (ABA) and silver nitrate (AgNO_3_). The maximum average number (35.96 ± 3.68) of mature SE was noticed on MS medium supplemented with 1.5 mg L^−1^ ABA. Mature embryos had two well-developed cotyledons and an elongated hypocotyl root axis. The development of SE into plantlets was significant for embryos matured on the medium with AgNO_3_ and ABA, with 86.67% and 83.33% conversion on the medium with 0.20 mg L^−1^ 6-benzylaminopurine (BAP). The plantlets thus produced acclimatized in a growth chamber before being transferred to the field, which showed 89.89% survival. The plants were morphologically similar to the mother plant with successful flowering.

## 1. Introduction

*Viola canescens* Wall. ex, Roxb., (family Violaceae) is an important endangered Himalayan herb as reported by the International Union for Conservation of Nature and Natural Resources. In India, it is known by different names in different regions: Banfsha or Vanaksha in Jammu and Kashmir; Kauru, Vanafsha or Banfasha in Uttarakhand; and Ratmundi, Gugluphul and Banfsaha in Himachal Pradesh [1,2,3]. The plant is one of the preferred herbs and used in both codified (Ayurveda, Unani, Siddha, homeopathy) and non-codified (folk) medicinal systems. Traditionally, it is used to cure a number of common ailments (e.g., cold, cough and sore throat) and different life-threatening diseases such as jaundice, renal problems, cancer and respiratory tract problems, and is an immune booster and blood purifier [1,4,5,6,7,8,9,10]. The plant is also known to have different pharmacological activities such as antimalarial [11], antifungal [12] and antibacterial activities [13,14,15,16]. Solvent extracts of *V. canescens* also showed relevant antioxidant and hepatoprotective activities [17].

Somatic embryogenesis is one of the promising plant biotechnology tools to produce identical individuals and is a preferred choice for fast plant regeneration. The use of somatic embryogenesis cuts the cost of propagation because there is no requirement of cutting and inoculating the shoots into a rooting medium for further rhizogenesis. Besides this, for long-term maintenance of in vitro raised tissue (calli) or shoots, repeated subculturing is required. Somatic embryos (SE), on the other hand, have the ability to withstand long-term storage via cryopreservation. Development of a protocol for somatic embryogenesis may also allow the embryos to be stored as synthetic seeds and used when required.

The lack of organized cultivation strategies, high ethnomedicinal and pharmacological potential, habitat preference and illegal indiscriminate collection from the wild have resulted in depletion of this plant from its wild habitat at an alarming rate. In a continuation of our previous work on the plant [18,19,20], an attempt was made to regenerate plant via somatic embryogenesis, which may contribute to mass production of the plant by the tissue culture method to fill the demand and supply gap of this medicinally important plant. Successful regeneration of plants via somatic embryogenesis has been reported in a number of Himalayan herbs such as *Podophyllum hexandrum* [21], *Picrorhiza kurroa* [22], *Swertia chirata* [23], *Angelica glauca* [24], *Gentiana kurroo* [25] and *Viola odorata* [26]. Since no previous work has been reported, to the best of our knowledge, on somatic embryogenesis in *Viola canescens,* the present work was conducted to develop an efficient regeneration protocol for *Viola canescens* through somatic embryogenesis. During this study, various factors were optimized to provide a protocol that may support its commercial production.

## 2. Material and Methods

### 2.1. Plant Material and Surface Sterilization

The leaves of *Viola canescens* were collected from its natural habitat located at latitude 30°8′59″ N and longitude 78°49′4″ E in the Nag Dev Hills of the Garhwal (Pauri) Himalayas. Collected plants from a single genotype were initially treated with lukewarm water with 2–3 drops of liquid soap with constant stirring for 10 min and repeating the same for 5 more mins by adding 2 drops of Tween-20. Finally, the flask was placed under running tap water for 15 min. Surface sterilization of the explants was carried out in a laminar air chamber; a 0.1% mercury chloride solution *w*/*v* was used for 90 s and then washed repeatedly with double-distilled autoclaved water to remove all traces of mercury chloride.

### 2.2. Culture Medium and Culture Conditions

Surface sterilized leaf explants were then blotted dry and prepared by trimming with the help of a scalpel and inoculated in the callus inducing medium [18,19]. Murashige and Skoog (MS) [19] medium supplemented with 3% (*w*/*v*) sucrose (Hi Media, India), 0.8% (*w*/*v*) agar (Hi Media, India), 1.5 mg L^−1^ 2,4-dichlorophenoxy acetic acid (2,4-D) and 1.5 mg L^−1^ Kinetin (Kn) was used as a culture medium. The pH of the medium was adjusted to 5.8 ± 0.2 prior to autoclaving. All cultures were maintained at 25 ± 2 °C temperature with a 16/8 hr photoperiod at 1000 lux light, and the humidity of the culture room was maintained at 60–70%.

### 2.3. Standardization of Somatic Embryogenesis

#### 2.3.1. Induction of SE

The embryogenic callus mass from leaf calli (300 mg) for somatic embryogenesis was transferred to MS medium with 2,4-D (0.10–0.25 mg L^−1^), indole-3-butyric acid (IAB) (0.10 –0.25 mg L^−1^), Kn (0.10–0.50 mg L^−1^) and 2,4-D+Kn (0.10 + 0.05; 0.15 + 0.05; 0.20 + 0.05 and 0.25 + 0.05 mg L^−1^) for 6 weeks. The induction frequency was calculated by using the equation “number of cultures showing SE embryos divided by total culture inoculated”. Each experiment was done in triplicate.

#### 2.3.2. Abscisic Acid and Silver Nitrate

Twelve-week-old callus mass (300 mg; 8 weeks on the culture medium + 4 weeks on the embryo induction medium) was used to study the effect of abscisic acid (ABA) (0.5–2.0 mg L^−1^), silver nitrate (AgNO_3_) (1.0–3.0 mg L^−1^) and growth regulator-free MS medium for SE maturation for 30 days. Both solutions were prepared fresh and the ABA solution was used after filter sterilization. Each experiment was done in triplicate.

#### 2.3.3. Germination of SE

Mature (cotyledon stage) SE from both treatments ABA and AgNO_3_ were transferred to MS medium supplemented with different concentrations of 6-benzylaminopurine (BAP) (0.10–0.50 mg L^−1^) and Kn (0.10–0.50 mg L^−1^). MS medium without growth regulators was used as a control. In total, 48 SE were inoculated in the control medium, and 16 and 20 mature SE with Kn and BAP, respectively, which was repeated thrice. Germination percentage was determined by the number of mature embryos germinated divided by the number of mature SE taken from their respective treatments. Each experiment was done in triplicate.

#### 2.3.4. Use of l-Glutamine and Casein Hydrolysate for Induction and Germination of SE

To study the effect of l-glutamine (50–200 mg L^−1^), casein hydrolysate (1–4% *w*/*v*) and casein + l-glutamine (1% + 50 mg L^−1^; 2% + 50 mg L^−1^ and 2%+ 100 mg L^−1^) for the further enhancement of somatic embryogenesis and regeneration of SE, equal amounts (300 mg) of 8-week leaf-derived callus was transferred to modified MS medium (MS medium with 2,4-D 0.15 mg L^−1^ + Kn 0.05 mg L^−1^). The mature SE (20 per culture tube) were then inoculated on the culture medium (induction medium) to observe the germination potential of the same medium (2,4-D and Kn was removed from cultures during embryo germination).

### 2.4. Acclimatization

In vitro raised complete elite plantlets were removed from the cultures and washed gently with distilled water to remove agar without damaging the delicate root system. Plantlets then transferred to (i) control garden soil (ii) forest soil + organic compost + sand (1:1:1 *v/v*) and (iii) rhizospheric forest soil + organic compost (2:1 *v/v*). The plantlets were initially placed in an environmental chamber under controlled conditions (1500 lux light for 3 days, followed by 2000 lux for the next 3 days and finally at 2500 lux light for the remaining days) under a 16 h photoperiod for 2 weeks and then diffuse light, and were irrigated with a dilute nutrient solution (every third day). The temperature of the environment chamber was maintained at 21 °C ± 1 °C. A dome made from a polythene bag with small holes (0.5 mm diameter) or sometimes glass beakers, as per requirements, was also used. The survival percentage was computed for the successfully established plants. The hardened plants were then transferred to earthen pots under environmental conditions. Plants were irrigated frequently and their growth and variation were monitored periodically.

### 2.5. Data Analysis

All the cultures were examined periodically and the data were pooled from 3 independent experiments. The effect of different treatments was analyzed using one-way analysis of variance (ANOVA) and the difference between their means was compared using Duncan’s Multiple Range Test (DMRT), a post hoc test, at *p* < 0.05. All the analyses were carried out using SPSS 16.0.

## 3. Results and Discussion

### 3.1. Somatic Embryo Induction

Callus was initiated from the leaf of *V. canescens* [18]. The generated callus was sub-cultured on a selected concentration of 2,4-D and Kn (1.5 + 1.5 mg L^−1^) for 8 weeks. These 8-week-old embryogenic calli were then inoculated on MS medium for somatic embryogenesis responses, and 2,4-D, IBA and Kn were used to induce somatic embryogenesis. Different concentrations of 2,4-D and IBA (0.10–0.25 mg L^−1^) were used to induce somatic embryogenesis. The results for both growth regulators remained significant (*p* ≤ 0.5), with 2,4-D proving superior over the IBA for both somatic embryo induction as well as the number of SE per clump. The maximum frequency (77.77%) and number of SE (17.55 ± 2.90) formed on the medium supplemented with 0.15 mg L^−1^ 2,4-D. The superiority of 2,4-D for somatic embryogenesis has been reported for *Foeniculum vulgae* [27], *Bunium persicum* [28], *Chlorophytum borivilianum* [29] and *Sapindus mukorossi* [30]. Besides this, the results showed profuse rooting in those callus clumps which were inoculated on MS medium supplemented with 0.10 mg L^−1^ 2,4-D, while beyond 0.15 mg L^−1^, the potential of 2,4-D and IBA for SE ceases. Different concentrations of Kn (0.10–0.50 mg L^−1^) were also evaluated to induce somatic embryos. The maximum frequency (55.55%) and average number (13.11 ± 2.29) were reported when the culture medium was supplemented with 0.20 mg L^−1^ and 0.25 mg L^−1^ concentrations, respectively. Further, beyond 0.25 mg L^−1^ concentration, shoot formation was reported from the inoculated callus. Cytokinin alone can produce SE, as reported in *Sapindus mukorossi* [30], *Corydalis yanhusuo* [31] and *Oncidium* [32]. However, in most cases, cytokinin is supplemented in MS medium with auxin for better results. The role of 2,4-D in SE induction has been well documented in various plant species [33,34], and the molecular mechanism, acting in particular through the modulation of endogenous hormonal levels, has been recently reviewed [35]. The present study is also in agreement with the early finding: the maximum induction frequency (88.88%) and average number of SE (19.15 ± 2.66) were reported when the 2,4-D + Kn (0.15 + 0.05) combination was tried (Table 1).

After selecting the optimum concentration of 2,4-D and Kn for somatic embryo induction, the selected concentration was tried with l-glutamine, an amino acid, in a concentration range of (50–200 mg L^−1^) and casein hydrolysate (1–4% *w*/*v*) and their combinations for further enhancement of somatic embryogenesis. Addition of both gave significant results as compared with the control (Table 2).

During study, it was further noticed that development of SE beyond the globular stage was inhibited in cultures with auxins (IBA and 2,4-D) and cytokinin (Kn). The inhibitory effect of auxin on maturation was reported by number of workers [36,37,38,39,40]. However, when l-glutamine, casein hydrolysate and their combinations were used (Table 2), the culture medium showed maturation of SE, but the SE were asynchronous and different stages of embryogenesis could be seen in the same culture, coupled with a slow maturation rate. The maximum average number of SE (27.66 ± 2.67) and maximum mature embryos (13.16 ± 3.48) were reported in modified MS medium supplemented with a combination of casein hydrolysate and l-glutamine (2 % + 50 mg L^−l^), respectively. The SE were then regenerated on the same medium (but without 2,4-D and Kn) in which they matured. The maximum average germination percentage was reported in MS medium supplemented with (2 % + 50 mg L^−l^) casein hydrolysate and l-glutamine. The addition of l-glutamine and casein hydrolysate, as sources of amino acid, to enhance somatic embryogenesis has been reported [33,34,35,36,37,38,39,40,41,42,43,44].

### 3.2. Maturation of SE

To enhance this rate of maturation, early SE were transferred to a medium without growth regulators for their maturation and supplemented with AgNO_3_ (1.0–3.0 mg L^−1^) and ABA (0.5–2.0 mg L^−1^). The use of silver nitrate improved the rate of SE maturation, with the maximum average number of SE (22.81 ± 3.85) observed when 1.0 mg L^−1^ was supplemented in the medium. However, ABA proved more suitable for SE maturation, with an average of 35.96 ± 3.68 mature SE observed in the MS medium supplemented with 1.5 mg L^−1^ ABA. Addition of AgNO_3_ and ABA improved maturation has been reported in carrot SE [45], *Phoenix dactylifera* [46], *Psidium guajava* [47] and *Chlorophytum borivilianum* [48], in agreement with the present work. The action of ABA on maturation has been well-described for both embryos and SE [35], acting through complete transcriptional reprogramming involving master regulators such as ABI3 (ABA Insensitive 3) [49,50,51]. Ethylene may antagonize ABA action during maturation [45,52]. The use of AgNO_3_, an inhibitor of ethylene biosynthesis, may help in obtaining effective maturation [45]. The action of AgNO_3_ has been reported to act through its inhibition of arginine decarboxylase in ethylene biosynthesis [45].

### 3.3. SE Germination

The mature SEs derived from three different treatments (i.e., SEs matured on the control (MS medium without growth regulators), the medium with ABA and the medium with AgNO_3_) (Table 3). These embryos were regenerated on MS medium, and MS medium supplemented with BAP (0.10–0.50 mg L^−1^) and Kn (0.10–0.50 mg L^−1^). An experiment was conducted to analyze the interaction of ABA and AgNO_3_ with SE and their rate of conversion into plantlets. MS medium with (Kn and BAP) or without cytokinin was used for the conversion of mature SEs into plantlets (plantlets with a minimum of one shoot and one root). Mature SE were transferred to MS to produce plantlets because the mature SE could accumulate all the proteins and enzymes necessary to germinate into plantlets.

**Figure 1:**
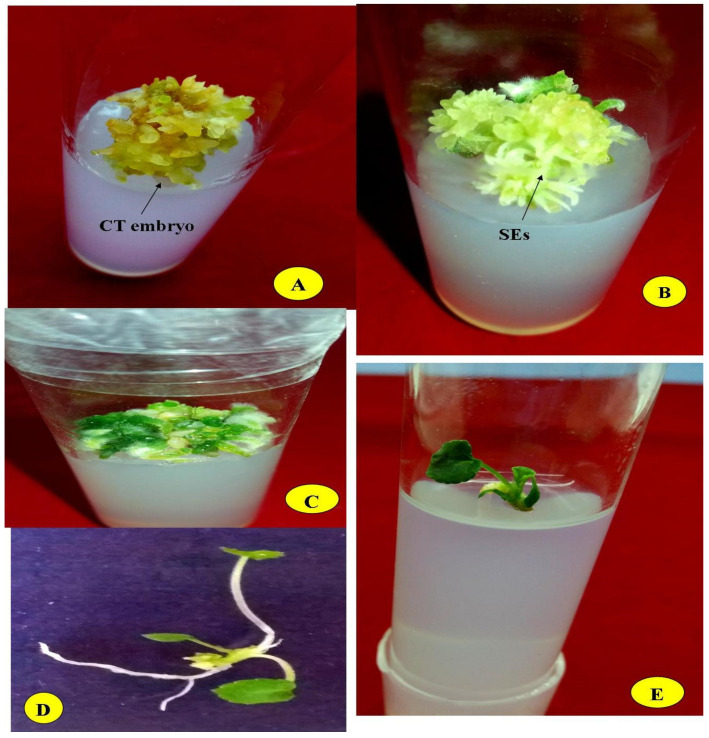
Plant regeneration through somatic embryogenesis in *V. canescens* from leaf explants. (**A**) Asynchronous SE on Murashige and Skoog (MS) medium 0.15 + 0.05 mg L^−1^ (2,4-D + Kn). (**B**) SE on MS medium supplemented with casein hydrolysate + l-glutamine (2 % + 50 mg L^−1^). (**C**) Mature SE. (**D**,**E**). Plantlets regenerated from mature SE.

There was 47.91% and 52.08% conversion of plantlets from mature embryos with ABA and AgNO_3_, respectively, in the MS medium without growth regulators, which is in agreement with the results of a number of workers [22,53]. The plantlet conversion from embryos matured on media with two different agents (ABA and AgNO_3_) was significant when BAP and Kn were tested. The maximum average conversion frequency (86.66%) was recorded when the MS medium was supplemented with 0.10 mg L^−1^ BAP for the SE matured on the medium with AgNO_3_ as an agent of maturation; 83.33% plantlet formation was reported for embryos matured on the medium with ABA. The two cytokinins, BAP (0.10–0.50 mg L^−1^) and Kn (0.10–0.50 mg L^−1^), strongly promoted plantlet formation when compared with the control (Table 4, Figure 1).

SE conversion declined in higher concentrations of both cytokinins, as previously reported [28,29,54,55,56]. Higher concentrations of both plant growth regulators (PGRs) supported organogenesis (shoot formation only) from the mature embryos and resulted in a decline in the conversion frequency in the present study.

### 3.4. Acclimatization

**Figure 2:**
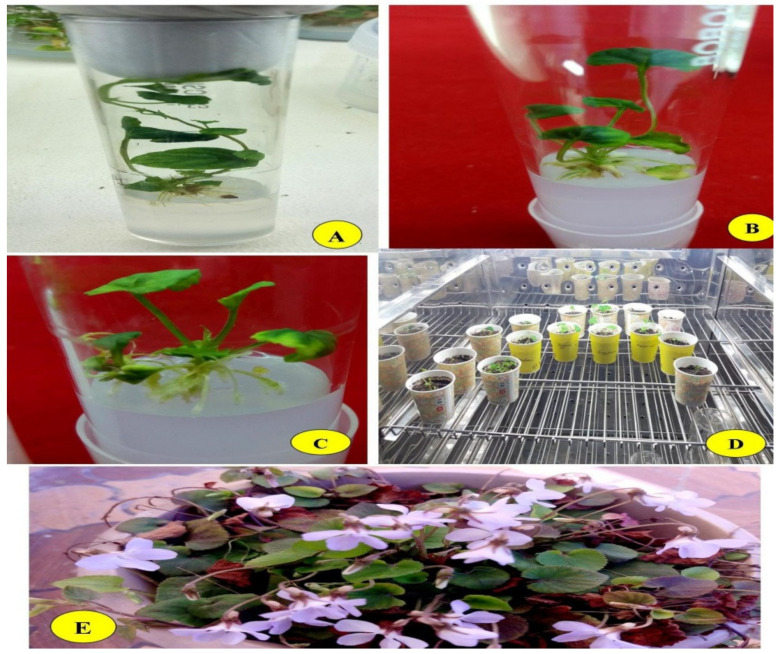
Regenerated *V. canescens* plants on MS medium (**A**–**C**). Plantlet acclimatization in the growth chamber (**D**). Representative 1-year-old established plantlet at the flowering stage (**E**).

The success of a plant tissue culture protocol depends upon the rate of plantlets survived outside the laboratory, because of a high mortality rate during the lab to field transfer [57,58,59]. The plantlets produced experimentally during the present study were acclimatized by keeping them in a growth chamber for 2 weeks. The plantlets were planted initially in thermocol cups and paper cups filled with sterilized soil (rhizospheric soil + organic compost at a 2:1 *w*/*v* ratio and forest soil + organic compost + sand at a 1:1:1 *v/v* ratio). The cups were then placed in a growth chamber under controlled conditions and watered regularly with 1/10 strength MS medium every third day before transferring the plants to earthen pots. Survival percentage was computed by counting the plants producing new leaves, and the results were significantly affected by the soil composition and acclimatization conditions (Table 5, Figure 2). The plantlets maintained in the control showed zero survival percentage, and the maximum percentage 88.89% was reported for the plantlets planted in rhizospheric soil.

## 4. Conclusions

The present paper is the first report on somatic embryogenesis in *V. canescens* to the best of our knowledge, as there are no previous reports of such studies so far for this medicinal plant. In the present study, the initiation of SE from leaf-derived calli in *V. canescens* was successfully achieved. The study focused on establishing the protocol for the production of high-quality SE; subsequently, the healthy plantlets had a high survival percentage. Maturation and germination of SE was worked out in detail. The addition of l-glutamine and casein hydrolysate to the MS medium promoted somatic embryo induction and maturation, while silver nitrate may be used for maturation of SE. The mature embryo showed a good rate of conversion when supplemented with BAP. Thus, the present protocol may be used to produce plants at a larger scale to meet the gap of demand and supply, and, at the same time, for its conservation. Finally, the application of the same protocol could be evaluated for in situ and ex situ conservation strategies in other endangered species of the genus *Viola* [60,61,62] at a global level.

## Figures and Tables

**Table 1:** Effect of PGRs on somatic embryogenesis in leaf-derived 8-week calli of *V. canescens* after 6 weeks of culture.

Concentration (mg L^−1^)				Response	Frequency of Embryogenesis	Number of SE Derived from 300 mg Callus
2,4-D	IBA	Kn	2,4-D + Kn			
0.10				RT	n.d.	n.d.
0.15				SE	77.77	17.55 ± 2.90 ^b^
0.20				SE	59.26	15.14 ± 2.89^c^
0.25				CA	n.d.	n.d.
	0.10			CA	n.d.	n.d.
	0.15			SE	29.62	8.03 ± 2.06 ^f^
	0.20			SE	37.03	10.66 ± 2.46 ^e^
	0.25			CA	n.d.	n.d.
		0.10		CA	n.d.	n.d.
		0.15		CA	n.d.	n.d.
		0.20		SE	55.55	10.25 ± 2.26 ^e^
		0.25		SE	48.14	13.11 ± 2.29 ^d^
		0.50		SH	n.d.	n.d.
			0.10 + 0.05	CA	n.d.	n.d.
			0.15 + 0.05	SE	88.88	19.15 ± 2.66 ^a^
			0.20 + 0.05	SE	70.37	16.81 ± 3.02 ^b^
			0.25 + 0.05	SE	62.96	15.22 ± 3.01 ^c^

CA = Callus; SH = Shoots; SE = Somatic embryos; RT = Profuse rooting; PRGs: plant growth regulators; n.d., not detected. Data were polled from three independent experiments (n = 27), where the values represent means ± SD. Means followed by the same letter in the column are non-significantly different by Duncan’s Multiple Range Test (DMRT) (p = 0.05).

**Table 2:** Effect of l-glutamine and casein hydrolysate on somatic embryogenesis in 8-week calli of *V. canescens* grown on MS* medium.

Treatment	Concentration	Number of SE per Callus Clump	Mature SE	Germination Percentage (%) per 20 Mature SE
Control		19.33 ± 2.9	n.d.	n.d.
l-Glutamine (mg L^−1^)	50	18.94 ± 1.95^e^	n.d.	n.d.
	100	23.33 ± 2.76 ^cd^	9.05 ± 3.13 ^c^	20
	200	19.05 ± 2.66 ^e^	5.11 ± 3.54 ^e^	15
Casein (% *w*/*v*)	1	21.66 ± 2.80 ^d^	n.d.	n.d.
	2	25.88 ± 2.56 ^b^	12.16 ± 3.39 ^ab^	55
	3	18.89 ± 2.05 ^e^	7.33 ± 3.69 ^d^	45
	4	13.05 ± 3.53 ^f^	2.38 ± 1.97 ^f^	n.d.
Casein+ l-Glutamine	1 % + 50 mg L^−1^	21.61 ± 2.93 ^d^	10.61 ± 2.68^bc^	60
	2 % + 50 mg L^−1^	27.66 ± 2.67 ^a^	13.16 ± 3.48 ^a^	55
	2 % + 100 mg L^−1^	24.16 ± 2.57^bc^	10.83 ± 1.82^bc^	35

n.d.: not detected. Data are from three independent experiments (*n* = 18), where the values represent means ± SD. Means followed by the same letter in the column are non-significantly different by DMRT (*p* = 0.05). Data for germination percentage per 20 SE without growth regulators were recorded after 30 days. MS* = MS medium with 0.15 + 0.05 mg L^−1^ 2,4-dichlorophenoxyacetic acid (2,4-D) and kinetin (Kn).

**Table 3:** Effects of abscisic acid (ABA) and silver nitrate on the maturation of somatic embryos in *V. canescens* after 30 days on MS medium without PGRs.

Treatments	Concentration (mg L^−1^)	Number of Mature SE per Callus
Control		21.25 ± 2.48 ^ef^
ABA	0.5	24.81 ± 2.94 ^d^
	1.0	29.59 ± 3.52 ^b^
	1.5	35.96 ± 3.68 ^a^
	2.0	27.70 ± 2.85 ^c^
Silver nitrate	1.0	22.81 ± 3.85 ^e^
	2.0	19.59 ± 2.73 ^f^
	3.0	20.88 ± 3.27 ^f^

PRGs: plant growth regulators; Data were obtained from three independent experiments (*n* = 27), where values represent means ± SD. Means followed by the same letter in the column are non-significantly different by DMRT (*p* = 0.05).

**Table 4:** Effect of different PGRs on conversion of somatic embryos (SE) into plantlets in *V. canescens* after 30 days.

BAP (mg L^−1^)	Kn (mg L^−1^)	Number of SE Cultured	Response of SE	Frequency of Plantlet Formation in SE Matured on the Medium with ABA	Response of SE	Frequency of Plantlet Formation in SE Matured on the Medium with AgNO_3_
Control		48	PL	47.91	PL	52.08
0.10		60	PL	83.33	PL	86.67
0.20		60	PL	73.33	PL	81.67
0.35		60	MR	28.33	SH	n.d.
0.50		60	SH	n.d.	SH	n.d.
	0.10	48	PL	77.08	PL	81.25
	0.20	48	MR	43.75	PL	75.00
	0.35	48	SH	n.d.	MR	64.58
	0.50	48	SH	n.d.	SH	n.d.

PRGs: plant growth regulators; n.d. = not detected; PL = plantlets; SH = shoots MR = mixed results (plantlets and shoots).

**Table 5:** Acclimatization of in vitro-raised plantlets of *V. canescens*.

Number of Plantlets	Plantlet Type	Duration in Growth Chamber (Weeks)	Irrigated with	Grown in Shade (Time Period in Weeks)	Survival %
30	SE	0	Water	0	00.00
**Rhizospheric forest soil + organic compost (2:1 *v/v*)**					
45	SE	2	1/10 MS medium + water	2	88.89
**Forest soil+ organic compost + sand (1:1:1 *v/v*)**					
45	SE	2	1/10 MS medium + water	2	73.33

SE: somatic embryo.

## Data Availability

All the data are included in the present study.

## References

[1] Rana C.S., Sharma A., Kumar N., Dangwal L.R., Tiwari J.K. (2010). Ethnopharmacology of some important medicinal plants of Nanda Devi National Park (NDNP) Uttarakhand. India. Nat. Sci..

[2] Masood M., Arshad M., Asif S., Chaudhari S.K. (2014). *Viola canescens*: Herbal wealth to be conserved. J. Bot..

[3] Mann N., Khajuria A.K., Uniyal P.L., Lakhanpaul S. (2016). *Viola canescens*: A potent medicinal herb of Himalaya. Botanica.

[4] Hamayun M., Khan S.A., Sohn E.Y., Lee I.J. (2006). Folk medicinal knowledge and conservation status of some economically valued medicinal plants of District Swat, Pakistan. Lyonia.

[5] Abbasi A.M., Khan M.A., Ahmed M., Zafar M. (2010). Herbal medicines used to cure various ailments by the inhabitants of Abbottabad district, North West Frontier Province, Pakistan. Indian J. Tradit. Knowl..

[6] Hussain I., Bano A., Ullah F. (2011). Traditional drug therapies from various medicinal plants of central karakoram national park, Gilgit-Baltistan Pakistan. Pak. J. Bot..

[7] Kumar S., Chand G., Sankhyan P., Chaudhari M., Kumar V., Gupta V., Keshari B.B., Sundaresan S. (2013). Herbal folk remedies for curing various ailments in Lug Valley of district Kullu, Himachal Pradesh (NW Himalaya). Int. J. Ayurvedic Herb. Med..

[8] Rana P.K., Kumar P., Singhal V.K., Rana C. (2014). Uses of local plant biodiversity among the tribal community of Pangi Valley of district Chamba in cold desert Himalaya, India. Sci. World J..

[9] Razzaq A., Hadi F., Rashid A., Ibrar M., Ali U. (2015). Exploration of medicinal plants and their conservation status at higher altitude of district Shangla, Khyber Pakhtunkhwa, Pakistan. Am. Eurasian J. Agric. Environ. Sci..

[10] Ahmad K.S., Qureshi R., Hameed M., Ahmad F., Nawaz T. (2012). Conservation assessment and medicinal importance of some plants resources from Sharda, Neelum valley, Azad Jammu and Kashmir, Pakistan. Int. J. Agric. Biol..

[11] Verma G., Dua V.K., Agarwal D.D., Atul P.K. (2011). Antimalarial activity of Holarrhena antidysenterica and *Viola canescens*, plants traditionally used against malaria in the Garhwal region of north-west Himalaya. Malar J..

[12] Rawal P., Adhikari R., Tiwari A. (2015). Antifungal activity of *Viola canescens* against *Fusarium oxysporum* f. sp. lycopersici. Int. J. Curr. Microbiol. Appl. Sci..

[13] Prasad D. (2014). Antimicrobial activities of whole plant of *Voila canescens* and *Bauhinia variegate*. Biosci. Biotechnol. Res. Asia.

[14] Khajuria A.K., Bisht N.S., Kumar G. (2017). Synthesis of Zinc oxide nanoparticles using leaf extract of *Viola canescens* Wall. ex, Roxb. and their antimicrobial activity. J. Pharmacogn. Phytochem..

[15] Khajuria A.K., Bisht N.S., Manhas R.K., Kumar G. (2019). Callus mediated biosynthesis and antibacterial activities of zinc oxide nanoparticles from *Viola canescens*: An important Himalayan medicinal herb. SN Appl. Sci..

[16] Khajuria A.K., Negi A., Bisht N.S., Mayura V., Kandwal A. (2019). Green synthesis, characterization and antimicrobial activity of synthesized zinc oxide nanoparticles using root extract of *Viola canescens* Wall. ex. Roxb. Asian J. Chem..

[17] Khan M.A., Ahmad W., Ahmad M., Nisar M. (2017). Hepatoprotective effect of the solvent extracts of *Viola canescens* Wall. ex. Roxb. against CCl 4 induced toxicity through antioxidant and membrane stabilizing activity. BMC Complement Altern. Med..

[18] Khajuria A.K., Bisht N.S., Krishan R. (2017). Effect of 2, 4-D and cytokinins on callus induction in different explants of *Viola canescens* wall. Ex, Roxb. Plant Arch..

[19] Khajuria A.K., Bisht N.S. (2018). Indirect in vitro Regeneration of *Viola canescens* Wall. ex, Roxb. by using Leaf Calli. Plant. Tissue Cult. Biotech..

[20] Khajuria A.K., Chandra S., Manhas R.K., Bisht N.S. (2019). Effect of different PGRs on in vitro organogenesis in *Viola canescens* Wall. ex. Roxb. from petiole callus culture. Vegetos.

[21] Nadeem M., Palni L.M.S., Purohit A.N., Pandey H., Nandi S.K. (2000). Propagation and conservation of *Podophyllum hexandrum* Royle: An important medicinal herb. Biol. Conserv..

[22] Sharma S., Katoch V., Rathour R., Sharma T.R. (2010). In vitro propagation of endangered temperate Himalayan medicinal herb *Picrorhiza kurroa* Royle ex benth using leaf explants and nodal segments. J. Plant Biochem. Biotechnol..

[23] Jha T.B., Dafadar A., Chaudhuri R.K. (2011). Somatic Embryogenesis in *Swertia chirata* Buch. Ham. ex Wall.—A Multipotent Medicinal Herb. Asian J. Biotechnol..

[24] Bisht A.K., Bhatt A., Dhar U., Bhatt A., Dhar U. (2015). Note on somatic embryogenesis and synthetic seed production in *Angelica glauca*: A valuable medicinal plant of Himalaya. J. Med. Plants Res..

[25] Kaushal S., Sidana A. (2018). Somatic embryogenesis and plant regeneration from cell suspension cultures of *Gentiana kurroo* Royle. Ann. Plant Sci..

[26] Narayani M., Varsha M.S., Potunuru U.R., Beaula W.S., Rayala S.K., Dixit M., Chadha A., Srivastava S. (2018). Production of bioactive cyclotides in somatic embryos of *Viola odorata*. Phytochemistry.

[27] Ohga Y., Ono M., Furuno K. (1989). Somatic embryogenesis and plant regeneration from hypocotyls and cotyledons of *Angelica acutiloba* and *Foeniculum vulgare*. Rep. Kyushu Branch Crop Sci. Soc. Jpn..

[28] Wakhlu A.K., Nagari S., Barna K.S. (1990). Somatic embryogenesis and plant regeneration from callus cultures of *Bunium persicum* Boiss. Plant. Cell. Rep..

[29] Rizvi M.Z., Kukreja A.K., Bisht N.S. (2010). In vitro propagation of an endangered medicinal herb *Chlorophytum borivilianum* Sant. et Fernand. through somatic embryogenesis. Physiol. Mol. Biol. Plants.

[30] Singh R., Rai M.K., Kumari N. (2015). Somatic embryogenesis and plant regeneration in *Sapindus mukorossi* Gaertn. from leaf-derived callus induced with 6-benzylaminopurine. Appl. Biochem. Biotechnol..

[31] Sagare A.P., Lee Y.L., Lin T.C., Chen C.C., Tsay H.S. (2000). Cytokinin-induced somatic embryogenesis and plant regeneration in *Corydalis yanhusuo* (Fumariaceae)—A medicinal plant. Plant Sci..

[32] Chen J.T., Chang W.C. (2001). Effects of auxins and cytokinins on direct somatic embryogenesison leaf explants of Oncidium ‘Gower Ramsey’. Plant Growth Regul..

[33] Raghavan V. (2004). Role of 2, 4-dichlorophenoxyacetic acid (2, 4-D) in somatic embryogenesis on cultured zygotic embryos of *Arabidopsis*: Cell expansion, cell cycling, and morphogenesis during continuous exposure of embryos to 2, 4-D. Am. J. Bot..

[34] Chaudhary K., Prakash J. (2019). Effect of 2, 4-D and Picloram on Somatic Embryogenesis in *Carica papaya* var. P-7-9. Plant Tissue Cult. Biotechnol..

[35] Méndez-Hernández H.A., Ledezma-Rodríguez M., Avilez-Montalvo R.N., Juárez-Gómez Y.L., Skeete A., Avilez-Montalvo J., De-La-Peña C., Loyola-Vargas V.M. (2019). Signaling overview of plant somatic embryogenesis. Front. Plant Sci..

[36] Gray D.J., Conger B.V. (1985). Influence of dicamba and casein hydrolysate on somatic embryo number and culture quality in cell suspensions of *Dactylis glomerata* (Gramineae). Plant Cell Tissue Organ Cult..

[37] Varisai M.S., Wang C.S., Thiruvengadam M., Jayabalan N. (2004). In vitro plant regeneration via somatic embryogenesis through cell suspension cultures of horsegram [*Macrotyloma uniflorum* (Lam.) Verdc.]. In Vitro Cell. Dev. Biol. Plant..

[38] Ageel S., Elmeer P. (2011). Effects of casein hydrolysates and glutamine on callus and somatic embryogenesis of date palm (*Phoenix dactylifera*). N. Y. Sci. J..

[39] Elmeer K.E.S., Junaid A., Srivastava P.S., Sharma M.P. (2013). Factors Regulating Somatic Embryogenesis in Plants. Somatic Embryogenesis and Gene Expression. Somatic Embryogenesis and Gene Expression.

[40] Daniel M.A., David R.H.A., Caesar S.A., Ramakrishnan M., Duraipandiyan V., Ignacimuthu S., Al-Dhabi N.A. (2018). Effect of l-glutamine and casein hydrolysate in the development of somatic embryos from cotyledonary leaf explants in okra (*Abelmoschus esculentus* L. monech). S. Afr. J. Bot..

[41] Fitch M.M. (1993). High frequency somatic embryogenesis and plant regeneration from papaya hypocotyls callus. Plant Cell Tissue Organ Cult..

[42] Choi Y.E., Kim J.W., Yoon E.S. (1999). High frequency of plant production via somatic embryogenesis from callus or cell suspension cultures in *Eleutherococcus senticosus*. Ann. Bot..

[43] Kim Y.S., Lim S., Choi Y.E., Anbazhagan V.R. (2007). High frequency plant regeneration via somatic embryogenesis in *Podophyllum peltatum* L., an important medicinal plant for source of anticancer drug. Curr. Sci..

[44] Zavattieri M.A., Frederico A.M., Lima M., Sabino R., Arnholdt-Schmitt B. (2010). Induction of somatic embryogenesis as an example of stress-related plant reactions. Electron. J. Biotechnol..

[45] Roustan J.P., Latche A., Fallot J. (1990). Control of carrot somatic embryogenesis by AgNO_3_, an inhibitor of ethylene action: Effect on arginine decarboxylase activity. Plant Sci..

[46] Al-Khayri J.M., Al-Bahrany A.M. (2001). Silver nitrate and 2-isopentyladenine promote somatic embryogenesis in date palm (*Phoenix dactylifera* L.). Sci. Hortic..

[47] Rai M.K., Jaiswal V.S., Jaiswal U. (2009). Shoot multiplication and plant regeneration of guava (*Psidium guajava* L.) from nodal explants of in vitro raised plantlets. J. Fruit. Ornam. Plant Res..

[48] Rizvi M.Z., Kukreja A.K., Bisht N.S. (2012). Plant regeneration in *Chlorophytum borivilianum* Sant. et Fernand. from embryogenic callus and cell suspension culture and assessment of genetic fidelity of plants derived through somatic embryogenesis. Physiol. Mol. Biol. Plants.

[49] Corbin C., Decourtil C., Marosevic D., Bailly M., Lopez T., Renouard S., Doussot J., Dutilleul C., Auguin D., Giglioli-Guivarc’H N. (2013). Role of protein farnesylation events in the ABA-mediated regulation of the pinoresinol–lariciresinol Reductase 1 (LuPLR1) gene expression and lignan biosynthesis in flax (*Linum usitatissimum* L.). Plant Physiol. Biochem..

[50] Markulin L., Drouet S., Corbin C., Decourtil C., Garros L., Renouard S., Lopez T., Mongelard G., Gutierrez L., Auguin D. (2019). The control exerted by ABA on lignan biosynthesis in flax (*Linum usitatissimum* L.) is modulated by a Ca2+ signal transduction involving the calmodulin-like LuCML15b. J. Plant Physiol..

[51] Markulin L., Corbin C., Renouard S., Drouet S., Durpoix C., Mathieu C., Lopez T., Auguin D., Hano C., Lainé É. (2019). Characterization of LuWRKY36, a flax transcription factor promoting secoisolariciresinol biosynthesis in response to *Fusarium oxysporum* elicitors in *Linum usitatissimum* L. hairy roots. Planta.

[52] Rojas-Lorz L., Arrieta-Espinoza G., Valdez-Melara M., Pereira L.F.P., Gatica-Arias A. (2019). Influence of silver nitrate on somatic embryogenesis induction in Arabica Coffee (*Coffea arabica* L.). Braz. Arch. Biol. Technol..

[53] Arnold Von S., Sabala I., Bozhkov P., Dyachok J., Filonova L. (2002). Developmental pathways of somatic embryogenesis. Plant Cell Tissue Organ Cult..

[54] Kackar A., Bhat S.R., Chandel K.P., Malik S.K. (1993). Plant regeneration via somatic embryogenesis in ginger. Plant Cell Tissue Organ Cult..

[55] Yusuf N.A., Annuar M.S., Khalid N. (2011). Rapid micropropagation of *Boesenbergia rotunda* (L.) Mansf. Kulturpfl. (a valuable medicinal plant) from shoot bud explants. Afr. J. Biotechnol..

[56] Verma M., Bansal Y.K. (2012). Induction of somatic embryogenesis in endangered butterfly ginger *Hedychium coronarium* J. Koenig. Indian J. Exp. Biol..

[57] Hazarika B.N. (2003). Acclimatization of tissue-cultured plants. Curr. Sci..

[58] Deb C.R., Imchen T. (2010). An Efficient In vitro Hardening Technique of Tissue Culture Raised Plants. Biotechnology.

[59] Chandra S., Bandopadhyay R., Kumar V., Chandra R. (2010). Acclimatization of tissue cultured plantlets: From laboratory to land. Biotechnol. Lett..

[60] Perrino E.V., Silletti G.N., Erben M., Wagensommer R.P. (2018). *Viola cassinensislucana* (Violaceae), a new subspecies from Lucanian Apennine, southern Italy. Phyton.

[61] Slazak B., Sliwinska E., Saługa M., Ronikier M., Bujak J., Słomka A., Göransson U., Kuta E. (2015). Micropropagation of *Viola uliginosa* (Violaceae) for endangered species conservation and for somaclonal variation-enhanced cyclotide biosynthesis. Plant Cell Tissue Organ Cult..

[62] Ballard H.E., Sytsma K.J., Kowal R.R. (1999). Shrinking the violets: Phylogenetic relationships of infrageneric groups in *Viola* (Violaceae) based on internal transcribed spacer DNA sequences. Syst. Bot..

